# METTL14-mediated N6-methyladenosine modification of SOX4 mRNA inhibits tumor metastasis in colorectal cancer

**DOI:** 10.1186/s12943-020-01220-7

**Published:** 2020-06-17

**Authors:** Xiaoxiang Chen, Mu Xu, Xueni Xu, Kaixuan Zeng, Xiangxiang Liu, Bei Pan, Chenmeng Li, Li Sun, Jian Qin, Tao Xu, Bangshun He, Yuqin Pan, Huilin Sun, Shukui Wang

**Affiliations:** 1 General Clinical Research Center, Nanjing First Hospital, Nanjing Medical University, No. 68, Changle Road, Nanjing, 210006 China; 2 Department of oncology, Nanjing First Hospital, Nanjing Medical University, Nanjing, 210006 Jiangsu China; 3 School of Medicine, Southeast University, Nanjing, 210009 Jiangsu China; 4 Department of Laboratory Medicine, The Second Affiliated Hospital of Nanjing Medical University, Nanjing, 210011 Jiangsu China; 5 Jiangsu Cancer Personalized Medicine Collaborative Innovation Center, Nanjing, 210029 Jiangsu China

**Keywords:** Colorectal cancer (CRC), N6-methyladenosine(m6A), METTL14, SOX4, YTHDF2

## Abstract

**Background:**

Colorectal cancer (CRC) is one of the leading causes of tumor-related death worldwide, and its main cause of death is distant metastasis. Methyltransferase-like 14(METTL14), a major RNA N6-adenosine methyltransferase, is involved in tumor progression via regulating RNA function. The goal of the study is to uncover the biological function and molecular mechanism of METTL14 in CRC.

**Methods:**

Quantitative real-time PCR (qRT-PCR), western blot and immunohistochemical (IHC) assays were employed to detect METTL14 and SOX4 in CRC cell lines and tissues. The biological functions of METTL14 were demonstrated using in vitro and in vivo experiments. Chromatin immunoprecipitation (ChIP), Transcrptomic RNA sequencing (RNA-Seq), m6A-RNA immunoprecipitation sequencing (MeRIP-Seq), RNA immunoprecipitation and luciferase reporter assays were used to explore the mechanism of METTL14 action.

**Results:**

METTL14 expression was significantly downregulated in CRC and decreased METTL14 was associated with poor overall survival (OS). Both the univariate and multivariate Cox regression analysis indicated that METTL14 was an independent prognostic factor in CRC. Moreover, lysine-specific histone demethylase 5C(KDM5C)-mediated demethylation of histone H3 lysine 4 tri-methylation(H3K4me3) in the promoter of METTL14 inhibited METTL14 transcription. Functionally, we verified that METTL14 inhibited CRC cells migration, invasion and metastasis through in vitro and in vivo assays, respectively. Furthermore, we identified SRY-related high-mobility-group box 4(SOX4) as a target of METTL14-mediated m6A modification. Knockdown of METTL14 markedly abolished SOX4 mRNA m6A modification and elevated SOX4 mRNA expression. We also revealed that METTL14-mediated SOX4 mRNA degradation relied on the YTHDF2-dependent pathway. Lastly, we demonstrated that METTL14 might inhibit CRC malignant process partly through SOX4-mediated EMT process and PI3K/Akt signals.

**Conclusions:**

Decreased METTL14 facilitates tumor metastasis in CRC, suggesting that METTL14 might be a potential prognostic biomarker and effective therapeutic target for CRC.

**Graphical abstract:**

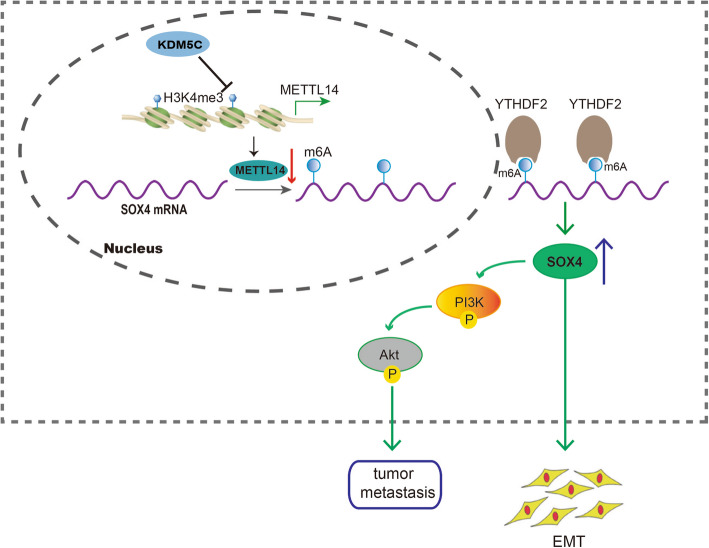

## Background

Colorectal cancer (CRC), one of the most common forms of malignancies in adults, ranks the third among leading causes of cancer-related death worldwide [1]. Due to the high rate of metastasis and recurrence, the mortality rate of CRC patients remains high [2]. Exploring the mechanisms underlying CRC progression will accelerate the search for the novel diagnostic biomarkers and the development of effective therapeutic target.

Analogous to histones and DNA, mRNAs can also be chemically modified [3]. More than 100 structurally distinct chemical modifications have been detected in RNAs [4, 5], among these, N6-methyladnosine(m6A) modification represents the most prevailing chemical mark in eukaryotic mRNAs [6, 7]. M6A modification is mainly mediated by the m6A methyltransferases (writers), including methyltransferase-like 14 (METTL14) [8], methyltransferase-like 3(METTL3) [9], vir-Like m6A methyltransferase associated (KIAA1429) [10] and Wilms tumor 1 associated protein (WTAP) [11], and can be removed by m6A demethylases (erasers) consists of alkylation repair homolog protein 5 (ALKBH5) [12] and fat-mass and obesity-associated protein (FTO) [13]. m6A modification exerts its effects on mRNAs via recruiting reader proteins, mainly including YTH domain-containing family protein 1/2/3(YTHDF1/2/3), insulin-like growth factor 2 mRNA-binding proteins 1/2/3(IGF2BP1/2/3) and heterogeneous nuclear ribonucleoprotein family (HNRNPA2B1, HNRNPC) [14, 15]. They are mainly involved in diverse biological regulatory processes, including RNA stability, translational regulation and primiRNA processing [16, 17].

Increasing studies have shown that m6A modification and its associated regulatory proteins play provital roles in the pathogenesis of varieties types of malignancies, including gastric cancer (GC) [18], hepatocellular cancer (HCC) [19], bladder cancer [20], breast cancer [21], lung cancer [22] and so on. However, the biological functions of m6A modification and knowledge of the mechanistic link among the m6A “writers”, “readers”, and “targets” remain largely elusive in CRC.

Our study unveiled that KDM5C-mediated demethylation of H3K4me3 lead to the inhibition of METTL14 in CRC. Moreover, we demonstrated the inhibitory role of METTL14 in CRC progression, and identified SOX4 as a downstream target of METTL14. Furthermore, METTL14 epigentically elevated SOX4 expression through a m6A-YTHDF2-dependent mechanism. Lastly, we found that inhibition of METTL14 in CRC promoted SOX4-mediated EMT process and activated SOX4-mediated PI3K/Akt signaling pathway. Taken together, we provide several new insights into METTL14-mediated m6A modification, and also uncover the molecular mechanism underlying CRC metastasis through identifying the downstream target genes and signals.

## Methods

### Analysis of public databases

The raw gene expression data in CRC were downloaded from The Cancer Genome Atlas (TCGA) (http://cancergenome.nih.gov) and GEO database. The independent data sets from (GSE9348 [23], GSE44076 [24–28], GSE41657) were analyzed in this study.

### Cell culture

Human normal colonial epithelial cell lines (NCM460) and colorectal cancer cell lines (HCT116, HCT8, SW620, SW480, HT29 and DLD-1) were all obtained from American Type Culture Collection (ATCC). NCM460, HCT116 and HCT8 cells were cultured in RPMI-1640 supplemented with 10% fetal bovine serum (FBS, Hyclone, USA), and SW620, SW480, HT29 and DLD-1 were maintained in Dulbecco’s modified Eagle’s medium (DMEM) with 10% FBS. All these cells were cultured at 37 °C with 5% CO2.

### Patients specimens and clinical data collection

A total of 136 CRC and corresponding adjacent normal tissues (ANTs) were collected from Nanjing First Hospital, Nanjing Medical Hospital. Our study was approved by the Institutional Review Board of Nanjing First Hospital, and written informed consent were obtained from all patients prior to our study. The patients who have achieved system treatment were not permitted in this study. The clinical characteristics in 136 CRC patients was presented in Table 1.

**Table 1:** Correlation between METTL14 expression and different clinical characteristics.

Characteristics	*n* = 136	METTL14 expression		*P* value
		high(%)(*n* = 68)	low(%)(n = 68)	
Gender				0.727
Male	81 (59.6%)	39 (57.4%)	42 (61.8%)	
Female	55 (40.4%)	29 (42.6%)	26 (38.2%)	
Age (years)				0.716
< 60	45 (33.1%)	24 (35.3%)	21 (30.9%)	
≥ 60	91 (66.9%)	44 (64.7%)	47 (69.1%)	
Tumor invasion depth				0.162
T1-T2	81 (59.6%)	45 (66.2%)	36 (52.9%)	
T3-T4	55 (40.4%)	23((33.8%)	32 (47.1%)	
Lymph node metastasis				0.018
N0	46 (33.8%)	30 (44.1%)	16 (23.5%)	
N1 + N2	90((66.2%)	38 (55.9%)	52 (76.5%)	
Distant metastasis				0.002
M0	117 (86.0%)	65 (95.6%)	52 (76.5%)	
M1	19 (14.0%)	3((4.4%)	16 (23.5%)	
TNM stage				0.005
I-II	83 (61.0%)	50 (73.5%)	33 (48.5%)	
III-IV	53 (39.0%)	18 (26.5%)	35 (51.5%)	

### Transwell assays

For transwell migration and invasion assays, CRC cells were seeded into the upper chamber without (transwell migration assay) or with (transwell invasion assay) matrigel (BD Biosciences, USA). After 24 h of incubation, non-migrated or invaded CRC cells were scraped off using a cotton swab, and CRC cells on the bottom of chamber were fixed with methanol for 10 min, and stained using 0.5% crystal violet. Then 5 fields(× 200 magnification) were selected and photographed randomly using an inverted microscope (Nicon, Japan). The experiments were performed in triple.

### Quantitative real-time PCR

TRIzol Reagent (Invitrogen, USA) was employed to extract total RNA from CRC tissues and cells following manufacturer’s instructions. The mRNA levels was assessed using PrimeScript RT reagent Kit and SYBR Premix Ex Taq (Takara, Dalian, China). All results were normalized to GAPDH. The relative expression of mRNAs was quantified using the 2^–∆∆Ct^ method. The primers used are listed in Additional file 1: Table S1.

### Plasmid construction and cell transfection

The full-length complementary cDNAs of human METTL14 and SOX4 were synthesized and cloned into the pcDNA3.1(Invitrogen, China). The small hairpin RNA (shRNA) targeting KDM5C, METTL14, SOX4, YTHDF1, YTHDF2 and YTHDF3 were designed and synthesized by GenePharma (Shanghai, China). The shRNA of SOX4, METTL14 and their negative control were synthesized and cloned into the pGLVH1/GFP/Puro vector (GenePharma, China). The plasmids were transfected into CRC cells using lipofectamine 3000(Invitrogen, USA) in accordance with the protocol. The sequences of shRNAs were supplemented in Additional file 1: Table S1. To achieved the METTL14 and SOX4 stable knockdown cell line, HCT116 cells were infected with LV-shMETTL14–1, LV-shSOX4 and LV-NC, and selected using 10 μg/ml puromycin.

### RNA stability

To measure RNA stability in METTL14 stable knockdown or control HCT116 cells, actinomycin D (MCE, USA) at 5 μg/ml was added to cells, and the cells were collected after incubation at the indicated times (0, 1, 2, 4, 8 h), and RNA was isolated from these cells for qRT-PCR.

### Chromatin immunoprecipitation assay

The chromatin immunoprecipitation (ChIP) assay kit (Beyotime, China) was employed to fulfill the ChIP assay following the manufacturer’s instruction. In brief, CRC cells were collected and soniacated to generate DNA fragments ranging from 200 to 500 bp. Then the lysate was immunoprecipitated with anti-KDM5C, anti-H3K4me3 or IgG antibodies (negative control) overnight. Immunoprecipitated DNAs were extracted and analyzed by qPCR. The 2000 bp upstream and 500 bp downstream of the METTL14 promoter were divided into eight parts(C1, C2, C3, C4, C5, C6, C7, C8), and the ChIP primer sequences were listed in Additional file 1: Table S3.

### Animal experiments

All animal experiments were approved by the animal care Committee of Nanjing First Hospital, Nanjing Medial University (acceptance No. SYXK 20160006). 2 × 10^6^ transfected HCT116 cells in 0.2 ml PBS were injected into the tail vein of nude mice which were randomly divided into nine groups (eight mice per group). After 2 months of injection, mice were sacrificed, and their lungs were removed and stained by Hematoxylin and Eosin (HE) Staining.

### Statistical analysis

All data analysis in our study were performed using GraphPad Prism 6(GraphPad, USA) and SPSS 18.0(SPSS, USA) software. Student’s t-test was employed to detect the differences in gene expression. A chi-square test was conducted to analyze the distribution differences of the variables, the Pearson correlation coefficient was employed to assess the correlation of expression. The survival curves were compared with log-rank test. Follow-up time was censored if the patient was lost to follow-up. Cox proportional hazards model was employed to perform univariate and multivariate analysis and calculate the 95% confidence interval (95% CI). *P* < 0.05 was considered statistically significant, data in our work are expressed as the mean ± standard deviation (SD) from more than three independent experiments.

A complete description of the methods, including Western blot, Immunohistochemistry (IHC) analysis, RNA m6A dot blot, RNA immunoprecipitation (RIP), RNA-Seq and MeRIP-Seq and Luciferase Reporter Assays are available in Additional file 2: supplementary materials and methods.

## Results

### METTL14 is low expressed in CRC and associated with CRC progression

**Fig. 1:**
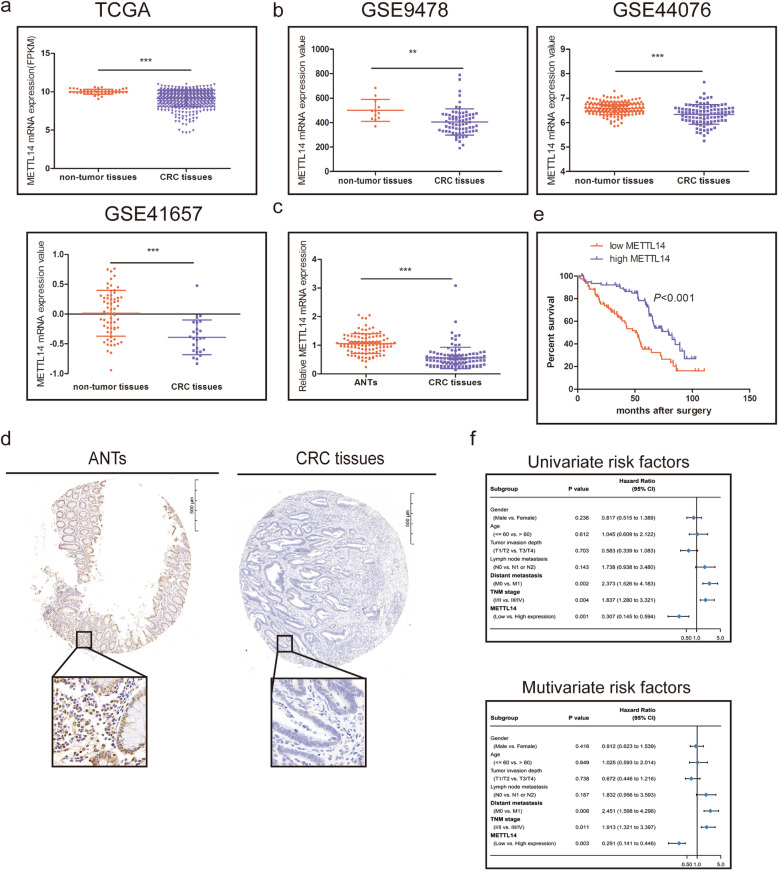
METTL14 expression is downregulated and is associated with prognosis in CRC. **a.** Expression of METTL14 in the TCGA CRC cohort. **b.** Expression of METTL14 in the GSE9348, GSE44076 and GSE41657 CRC cohorts. c. The METTL14 mRNA expression levels in 136 CRC tissues and matched normal tissues were detected using qRT-PCR. d. Representive images of IHC staining for METTL14 protein on a tissue microarray constructed from 136 CRC tissues and matched normal tissues. e.Kaplan-Meier OS analysis of METTL14 expression in CRC patients. f. Univariate and multivariable analyses were performed in the CRC cohort. All bars correspond to 95% CIs. **P < 0.01, ***P < 0.001

In order to detect the expression of METTL14 in CRC, we first sought to determine the METTL14 expression levels from TCGA database, the results showed that the expression of METTL14 was significantly downregulated in CRC tissues (Fig. 1a). We also complied the gene expression from GEO dataset (GSE9348, GSE44076, GSE41657), and confirmed that the METTL14 expression levels were decreased in CRC tissues (Fig. 1b). Moreover, METTL14 was examined by qRT-PCR and IHC on samples from 136 CRC patients compared with corresponding ANTs, and elevated METTL14 expression both on mRNA and protein levels were observed in CRC tissues (Fig. 1c, d). Furthermore, downregulated expression of METTL14 was significantly associated with Lymph node metastasis(*P* = 0.018), distant metastasis(*P* = 0.002) and TNM stage(*P* = 0.005) (Table 1). Survival analysis using Kaplan-Meier method indicated that CRC patients with low METTL14 expression exhibited a worse overall survival (OS) (Fig. 1e). Unvariate and multivariate analyses showed that METTL14 expression could be an independent prognostic for OS (Fig. 1f). Collectively, these results indicated that METTL14 was significantly downregulated in CRC and might be associated with CRC progression.

### KDM5C-mediated demethylation of H3K4me3 inhibited METTL14 transcription

**Fig. 2:**
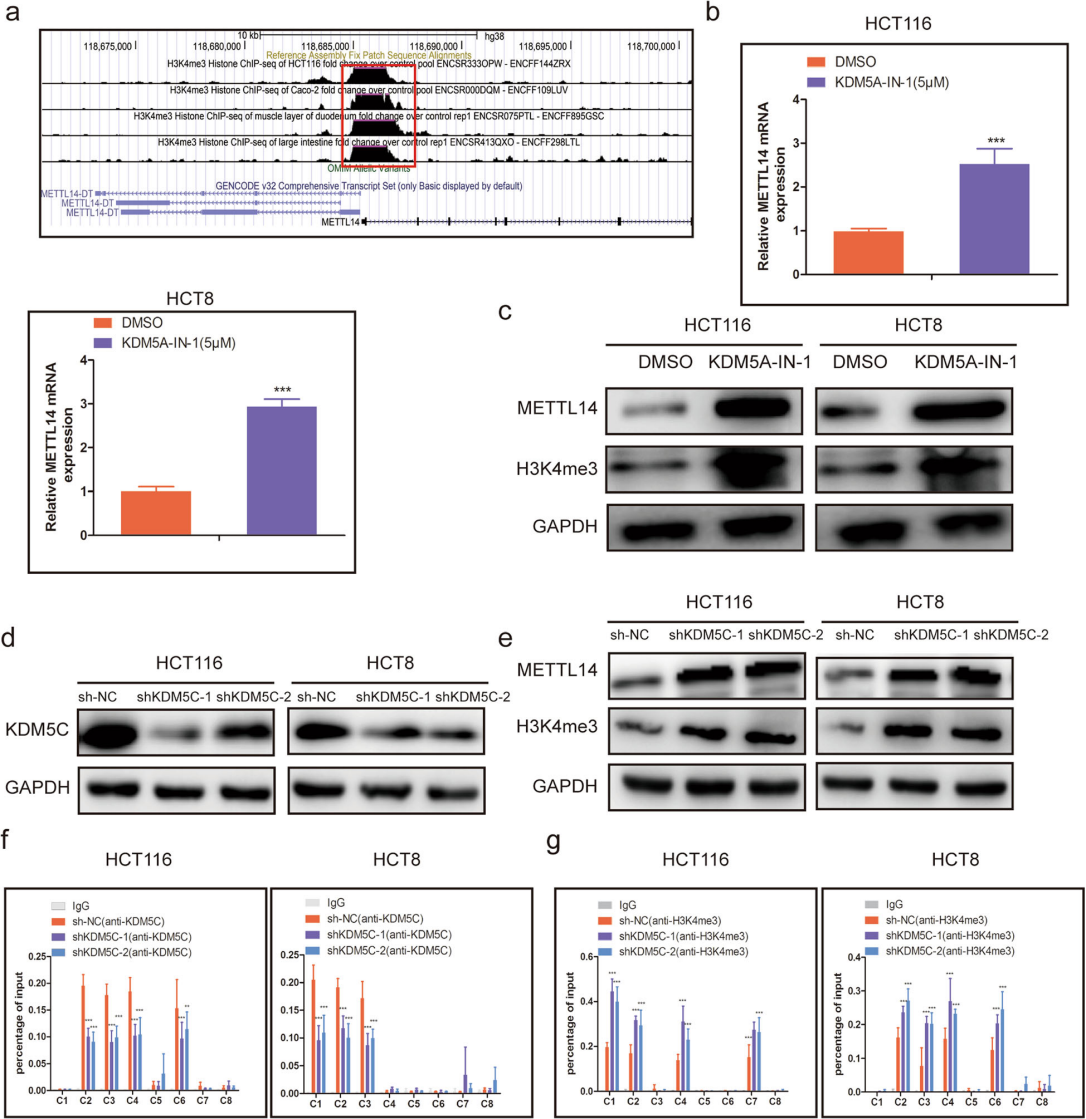
KDM5C-mediated demethylation of H3K4me3 inhibited METTL14 transcription. **a.** Analysis of H3K4me3 ChIP-Seq data of CRC cells and colorectal tissues in the METTL14 locus. b. The mRNA levels of METTL14 in KDM5A-IN-1(5 μM)-treated HCT116 and HCT8 cells were measured using qRT-PCR. **c**. The METTL14 and H3K4me3 protein levels in HCT116 and HCT8 cells were detected by western blot after KDM5A-IN-1(5 μM) treatment. **d**. The KDM5C knockdown efficiency was determined at the protein levels in HCT116 and HCT8 cells using western blot. **e**. The METTL14 and H3K4me3 protein levels in HCT116 and HCT8 cells were detected using western blot. f. ChIP assays were employed to measure the levels of KDM5C binding **f** and the enrichment of H3K4me3 **g** at the promoter of METTL14 in KDM5C deficiency or control HCT116 and HCT8 cells. ***P < 0.001

To uncover the mechanism of low METTL14 expression in CRC, we analyze the ChIP-Seq data of H3K4me3 in the Encyclopedia of DNA Elements (ENCODE) database, and found that the enrichment of H3K4me3 in the promoter region was markedly lower in CRC cells than that in normal colorectal tissues (Fig. 2a). KDM5C belongs to KDMs family, and could catalyze H3K4me2/3 demethylation and suppress gene transcription via decreasing H3K4 methylation [29, 30]. Using UALCAN database (http://ualcan.path.uab.edu/analysis.html), we found that KDM5C mRNA expression was markedly upregulated in CRC tissues (Additional file 3: Fig. S1a), in agreement with the results from TCGA database, KDM5C mRNA expression levels was elevated in our CRC cohort (Additional file 3: Fig. S1b), and the expression of METTL14 was negatively correlated with the KDM5C expression levels (Additional file 3: Fig. S1c). We then treated HCT116 and HCT8 cells with KDM5C inhibitor (KDM5A-IN-1), and found that both RNA and protein levels of METTL14 were significantly upregulated after the treatment (Fig. 2b, c). Moreover, we knocked down KDM5C, and found that KDM5C-deleption significantly elevated the expression levels of METTL14 (Fig. 2d, e). Furthermore, the ChIP results showed that the promoter of METTL14 was enriched in KDM5C binding and H3K4me3 signals, and knockdown of KDM5C could obviously increase the enrichment of H3K4me3 signals in the promoter of METTL14 (Fig. 2f, g). Our data indicated that KDM5C-mediated histone H3K4me3 loss in the promoter region might account for the decrease of METTL14.

### METTL14 inhibits CRC cell migration, invasion and metastasis

**Fig. 3:**
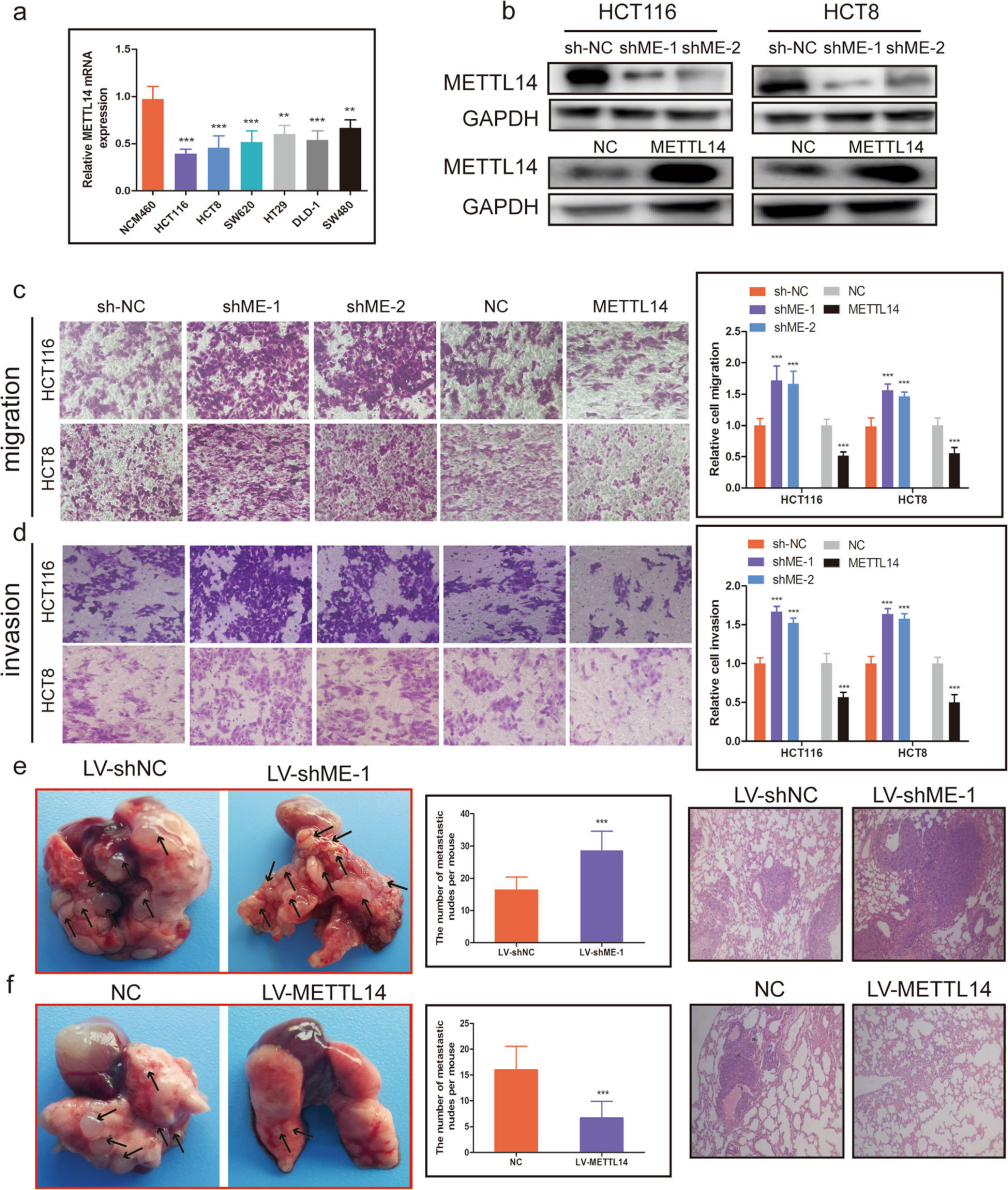
METTL14 inhibits CRC cell migration, invasion and metastasis. **a**. The expression of METTL14 in CRC cell lines (HCT116, HCT8, HT29, SW620, SW480 and DLD-1) compared with NCM460 detected using qRT-PCR. **b**. The protein levels of METTL14 in HCT116 and HCT8 cells with METTL14 knockdown or METTL14 overexpression were measured by western blot. **c**. Transwell migration assays were used to determine the migratoy abilities of HCT116 and HCT8 cells with METTL14 knockdown or overexpression. **d**. Transwell invasion assays were employed to detect the invasive abilities of HCT116 and HCT8 cells with METTL14 knockdown or overexpression. **e**. Left panel, representative images of metastatic nodes in the lungs from LV-shNC and LV-shME-1 groups. Middle panel, quantification of the metastatic nodes from LV-shNC and LV-shME-1 groups. Right panel, HE-stained lung sections from LV-shNC and LV-shME-1 groups. **f**. Left panel, representative images of metastatic nodes in the lungs from LV-NC and LV-METTL14 groups..Middle panel, quantification of the metastatic nodes from LV-NC and LV-METTL14 groups. Right panel, HE-stained lung sections from LV-NC and LV-METTL14 groups.**P < 0.01, ***P < 0.001

To investigate the function role of METTL14 in CRC cells, we first examine METTL14 expression levels in CRC cell lines, consistent with the results from CRC tissues, METTL14 was significantly downregulated in CRC cell lines compared with non-CRC cell line (Fig. 3a). Next, METTL14 was knocked down using two shRNA targeting METTL14(shME-1,shME-2), while overexpressed METTL14 using METTL14 expression vector (pcDNA3.1-METTL14) in HCT116 and HCT8 cells (Fig. 3b). Transwell migration assay revealed that forced expression of METTL14 apparently impeded the migratory ability of HCT116 and HCT8 cells, attenuation of METTL14 expression significantly elevated the migration speed of HCT116 and HCT8 cells (Fig. 3c). Correspondingly, transwell invasion assay showed that the invasive ability of HCT116 and HCT8 cells was significantly suppressed in response to METTL14 upregulation, while it was obviously enhanced by knockdown of METTL14 (Fig. 3d). Furthermore, we assessed the physiological relevance of METTL14 to CRC metastasis in vivo, stable cells with modified METTL14 expression were tail-vein injected into the BABL/c nude mice, after 8 weeks, HCT116 cells with METTL14 deficiency dramatically facilitated CRC cell metastasis (Fig. 3e), as evidenced by the number of lung metastatic lesions, in contrast, overexpression of METTL14 markedly inhibited CRC cell metastasis, as shown by the number of lung metastatic lesions (Fig. 3f).

### SOX4 is a downstream target of METTL14

**Fig. 4:**
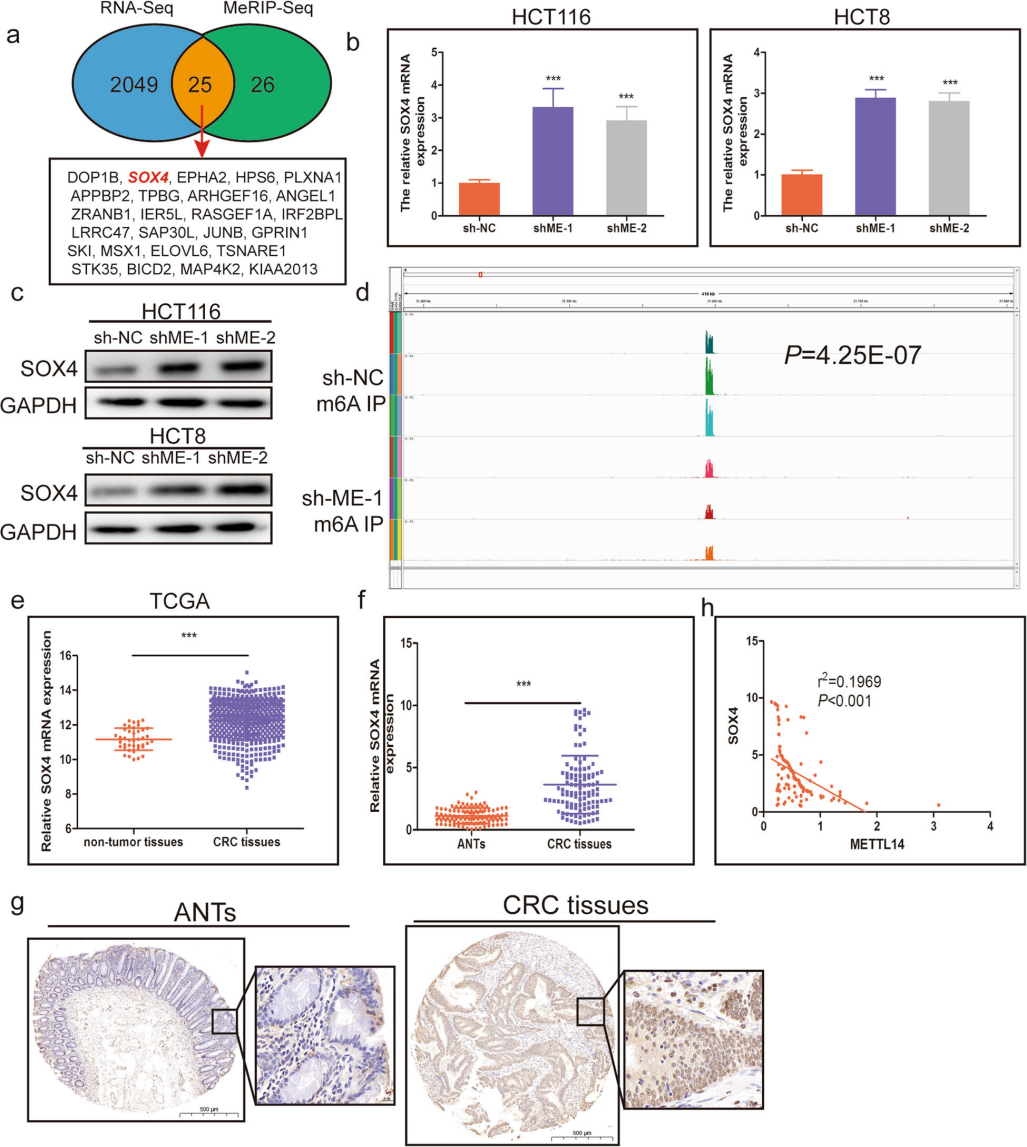
SOX4 is a downstream target of METTL14. **a**. MeRIP-Seq and RNA-Seq identidied differentially expressed genes in METTL14 stable knockdown cells when compared with their corresponding controls. **b, c**. The mRNA **b** and protein **c** levels of SOX4 in METTL14-deficient HCT116 and HCT8 cells were detected using qRT-PCR and western blot, respectively. **d**. Expression of SOX4 mRNA in the TCGA cohort. **e**. The METTL14 mRNA expression levels in 136 CRC tissues and matched normal tissues were detected using qRT-PCR. **f**. Representive images of IHC staining for SOX4 protein on a tissue microarray constructed from 136 CRC tissues and matched normal tissues. **g**. The correlation between METTL14 and SOX4 in CRC. ***P < 0.001

To identify the molecular mechanism by which METTL14 inhibits CRC metastasis, we used RNA-seq in CRC cells with stable METTL14 downregulation and conducted MeRIP-seq in CRC cells with stable METTL14 downregulation and control cells. RNA-seq data showed that 2074 transcripts were markedly upregulated on METTL14 knockdown (Additional file 4: Table S1). MeRIP-seq presented that m6A peaks of 51 transcripts exhibited decreased abundance (fold change<− 1, *P* < 0.05) (Additional file 4: Table S2). Intriguingly, 25 transcripts were overlapped in the transcriptome-sequencing and MeRIP-seq data (Fig. 4a). Of these candidate genes, SOX4 was reported be closely with tumor progression, and was selected as a candidate target of METTL14-mediated m6A modification for further study. SOX4 was significantly upregulated in METTL14-knockdown HCT116 and HCT8 cells both on mRNA and protein levels (Fig. 4b, c).

The m6A usually happens in RRACH(R = G or A, H = A, C or U) consensus sequence, our MeRIP-seq results showed that an m6A peak was detected around the stop condon of SOX4 mRNA in nontarget control shRNA HCT116 cells and were all decreased upon METTL14 knockdown (Fig. 4d). Therefore, a preliminary conclusion was drawn that SOX4 was a METTL14 downstream target.

Through analyzing the transcriptome data in TCGA database, we found that SOX4 mRNA was markedly upregulated in CRC tissues in comparison with normal tissues (Fig. 4e), similar result was also found in GSE9348, GSE44076 and GSE41657 (Additional file 3: Fig. S2). Our qRT-PCR and IHC results further confirmed the change (Fig. 4f, g). In addition, we also found that the expression of METTL14 was significantly reversely correlated with SOX4 expression (Fig. 4h).

### METTL14 knockdown enhances SOX4 mRNA stability via an m6A-YTHDF2-dependent pathway

**Fig. 5:**
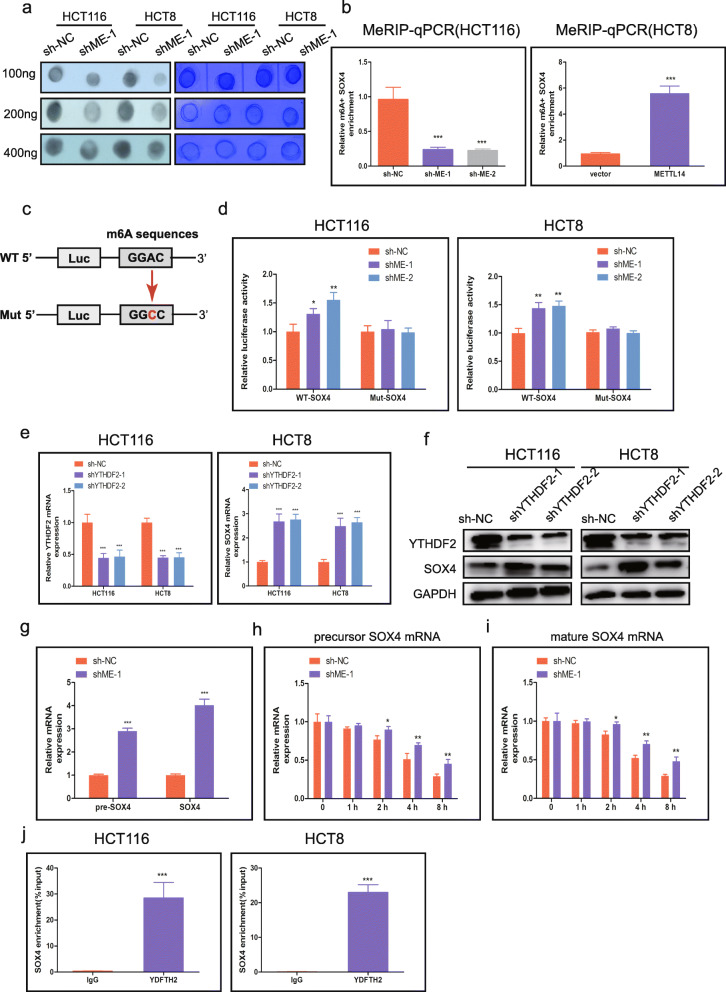
METTL14 knockdown enhances SOX4 mRNA stability via an m6A-YTHDF2-dependent pathway. **a.** The m6A contents of total RNAs in METTL14-knockdown HCT116 and HCT8 cells (left panel) were detected using dot blot with m6A antibody. Methylene blue staining were served as the loading control (right panel). **b**. MeRIP-qPCR analysis was used to demonstrate METTL14-mediated SOX4 m6A modification in HCT116 and HCT8 cells. m6A modification of SOX4 was depleted upon METTL14 knockdown. **c**. Wild-type or m6A consensus sequence mutant SOX4 cDNA was fused with firely luciferse reporter. **d**. Mutation of m6A consensus sequences or knockdown of METTL14 relieved the posttranscriptional repression of SOX4 in HCT116 and HCT8 cells. **e**. The mRNA levels of YTHDF2 and SOX4 in YTHDF2 knockdown CRC cells were detected by qRT-PCR. **f**. The protein levels of YTHDF2 and SOX4 in YTHDF2 knockdown CRC cells were detected by western blot. GAPDH was used as control. **g**. Precursor and mature mRNA of SOX4 in METTL14 stable knockdown and control HCT116 cells. h. i. The precursor **h** and mature **i** SOX4 mRNA expression were detected at indicated times. j. RIP-qPCR assay using YTHDF2-specifc antibody and IgG control antibody to measure the enrichment of YTHDF2 binding to SOX4 m6A modification sites. *P < 0.05, **P < 0.01, ***P < 0.001

To further verify that METTL14 targets SOX4 mRNA via m6A modification, we first detected the global level of m6A in control and METTL14 stable knockdown group through m6A dot blot assay (Fig. 5a). As expected, m6A levels were obviously reduced with the deletion of METTL14 in HCT116 and HCT8 cells. Then, MeRIP-qPCR was conducted to measure the enrichment of m6A in SOX4. Our results showed that the m6A abundance in the SOX4 mRNA was dramatically increased on METTL14 overexpression as well as substantially diminished on METTL14 knockdown (Fig. 5b). To further determine the effect of m6A modification on SOX4 expression, luciferase reporters containing either wild-type or mutant SOX4 to address the effect of m6A modification on SOX4 expression. For the mutant form of SOX4, the adenosine bases in m6A consensus sequences (RRACH) were replaced by cytosine, thus m6A modification was abolished (Fig. 5c). Luciferase reporter assay revealed that transcriptional level of wild-type SOX4, but not the mutation, obviously increased in the absence of METTL14 (Fig. 5d), revealing that the regulation of SOX4 level was the under control of METTL14-related m6A modification.

Recently, studies have reported that YTHDF1/2/3, a distinct family of m6A readers that could target thousands of mRNA via recognizing m6A motif [31]. Therefore, we explore the effect of YTHDF1/2/3 on SOX4 mRNA stabilisation. Two specific shRNAs targeting YTHDF1/2/3 were employed and western blot was used to confirm the knockdown efficiency (Fig. 5e, f). We found that YTHDF2 knockdown strongly augmented SOX4 expression, while YTHDF1/3 had no effect on SOX4 in HCT116 and HCT8 cells (Fig. 5e, f, Additional file 3: Fig. S3a,b). To further investigate the mechanism regulating SOX4 expression through m6A modification, we firstly detected the expression of precursor (pre) and mature (mat) mRNA of SOX4 in METTL14 stable knockdown and control HCT116 cells, and the results showed that both pre and mat mRNA expression levels were substantially enhanced in METTL14 stable knockdown cells (Fig. 5g). We then treated METTL14 stable knockdown and control HCT116 cells with actinomycin D to block transcription. Our results revealed that half-life of both precursor and mature mRNA in control cells were markedly shorter than in METTL14 stable knockdown cells (Fig. 5h, i). It indicated that m6A modification might trigger the splicing of precursor mRNA and the degradation of mature mRNA of SOX4. As shown in Fig. 5j, when compared to IgG control, YTHDF2-specific antibody substantially reduced the enrichment of SOX4 mRNA in the RIP assay. Above results confirmed that SOX4 was a target of YTHDF2. Moreover, we found that there was no differences in the expression of YTHDF2 between in CRC tissues and non-tumor tissues. (Additional File 3: Fig. S3c). The results showed that METTL14 couldn’t affect YTHDF2 expression levels, but could raise YTHDF2 to m6A modified sites of SOX4 mRNA, and modulated the expression of SOX4. Collectively, METTL14-mediated m6A modification repressed SOX4 expression through YTHDF2-dependent mRNA degradation.

### SOX4 served as an oncogene and reversed the effects of METTL14 in CRC

**Fig. 6:**
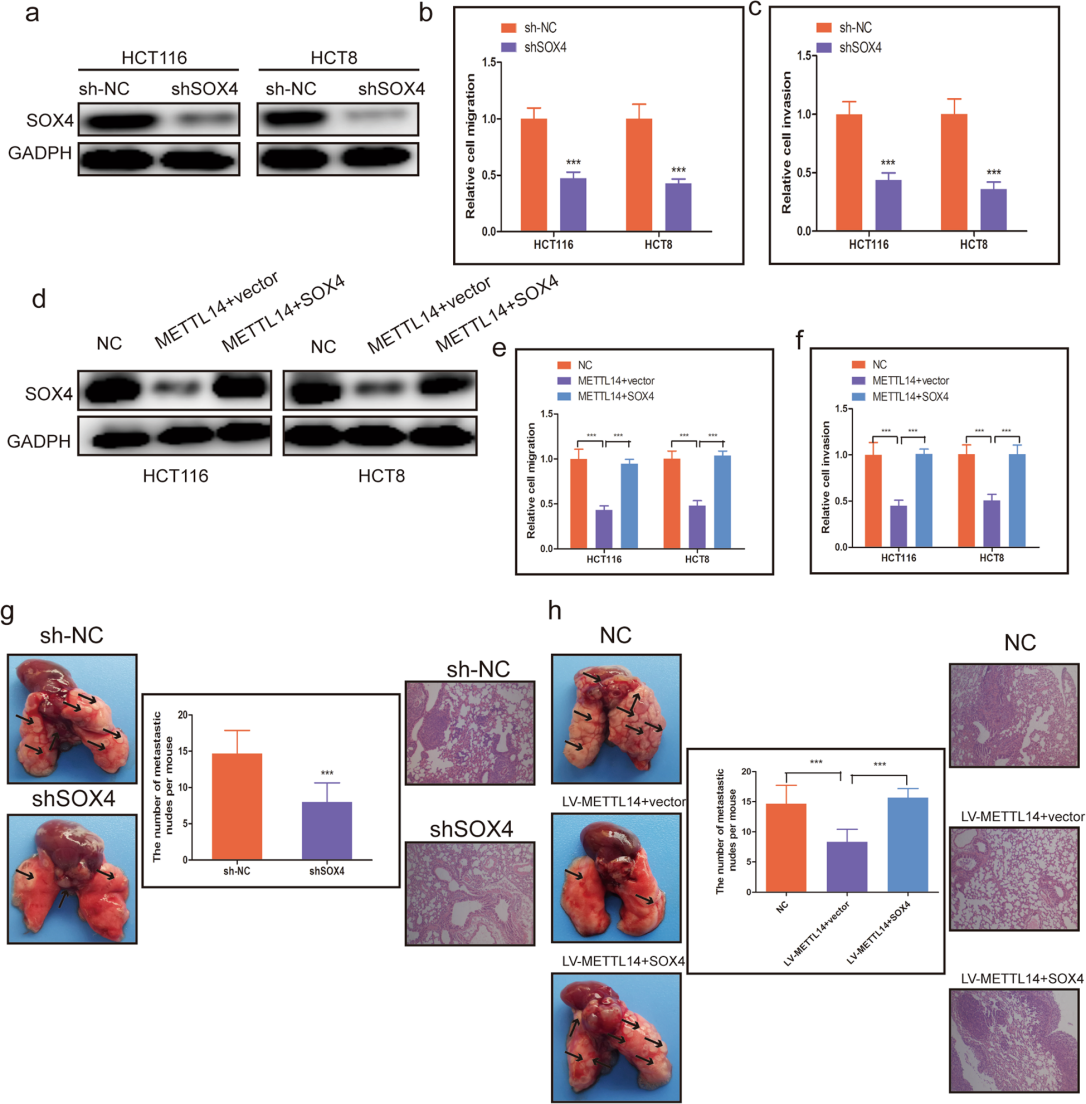
SOX4 served as an oncogene and reversed the effects of METTL14 in CRC. **a**. The SOX4 knockdown efficiency was proved at the protein levels in HCT116 and HCT8 cells by western blot assay. **b**. Quantified results of the cell migration abilities of HCT116 and HCT8 cells with SOX4 deficiency. **c**. Quantified results of the cell invasion abilities of HCT116 and HCT8 cells with SOX4 deficiency. **d**. The protein levels of METTL14 and SOX4 in HCT116 and HCT8 cells transfected with METTL14 and SOX4 expression plasmid or blank vector (vector) were measured using western blot. **e, f**. Quantified results of the cell migration **e** and invasion **f** abilities of cell migration abilities of METTL14-overexpressing HCT116 and HCT8 cells added with SOX4 expression plasmid or blank vector. **g**. Left panel, representative images of the gross lesion in the lung tissues from sh-NC and shSOX4 group. Middle panel, quantification of the metastatic nodes from indicated groups. Right panel, representative microscopic views of pulmonary metastatic foci from indicated groups using HE staining. **h**. Left panel, representative images of the gross lesion in the lung tissues from negative control (NC), LV-METTL14 + vector and LV-METTL14 + SOX4 group. Middle panel, quantification of the metastatic nodes from indicated groups. Right panel, representative microscopic views of pulmonary metastatic foci from indicated groups using HE staining. **P < 0.01, ***P < 0.001

To further investigate the oncogenic function of SOX4 in CRC, the expression of SOX4 in HCT116 and HCT8 cells was strikingly downregulated using shRNA targeting SOX4(shSOX4) (Fig. 6a). Then the loss-of-function experiments were employed, transwell migration assays revealed that SOX4 upregulation lead to increase CRC cell migration (Fig. 6b, Additional file 3: Fig. S4a), and transwell invasion assay strongly showed that SOX4 overexpression elevated the ability of CRC cell invasion (Fig. 6c, Additional file 3: Fig. S4b). To further validate the association between METTL14 and SOX4, we then studied the effects of METTL14 upregulation on cell migration and invasion after SOX4 overexpression, and found that the reduced migratory and invasive capabilities of HCT116 and HCT8 cells caused by METTL14 overexpression could be extensively retarded by SOX4 upregulation (Fig. 6d, e, f, Additional file 3: Fig. S4c, 4d). Mice inoculated with SOX4 stable knockdown HCT116 cells had less metastatic foci in the lungs than mice that received control cells, while there were fewer metastatic foci in the lungs of mice after injecting SOX4-silencing HCT-116 cells in comparison with control groups (Fig. 6g). In addition, SOX4 overexpression partially restored the metastasis capacity inhibited by METTL14 upregulation (Fig. 6h).

### METTL14 inhibits CRC malignant process through SOX4-mediated EMT process and PI3K/Akt signals

**Fig. 7:**
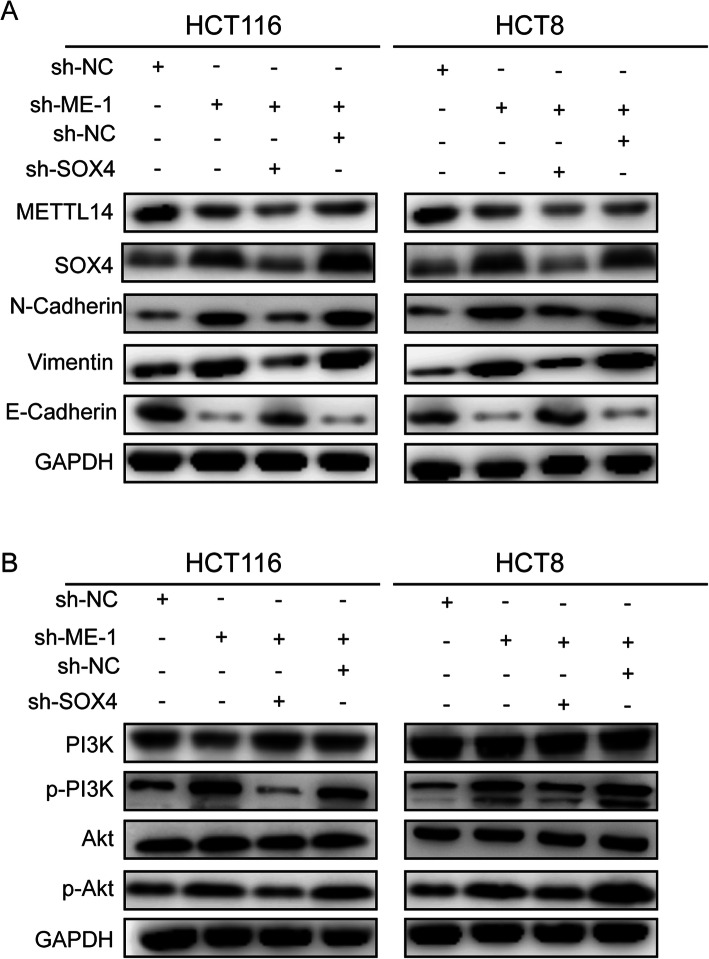
METTL14 inhibits CRC malignant process through SOX4-mediated EMT process and PI3K/Akt signals. **a**. Protein levels of METTL14, SOX4, E-cadherin, Vimentin and N-cadherin were detected by western blot in HCT116 and HCT8 cells with indicated treatment. **b**. Protein levels of PI3K, p-PI3K, Akt and p-Akt were detected by western blot in HCT116 and HCT8 cells with indicated treatment. GAPDH was used as a control

A number of studies have verified that SOX4 controlled the TGF-β-induced epithelial-to-mesenchymal (EMT) [14, 32], a process closely associated with increases in invasive and migratory capacity of tumor cells. In the present study, we found that depletion of METTL14 elevated the expression of N-cadherin and Vimentin as well as reduced E-cadherin expression levels, indicating that disruption of METTL14 promotes EMT process, while silencing of SOX4 could reverse this promotion caused by METTL14 knockdown (Fig. 7a). Moreover, It has been reported that SOX4 is an crucial activator of MAPK, PI3K-Akt and Wnt signaling [33]. Here, we found that METTL14 knockdown facilitated the phosphorylation of the PI3K and Akt proteins, indicating that METTL14 knockdown activated the PI3K/Akt signaling, and SOX4 silencing partially reversed the activation of the PI3K/Akt pathway induced by METTL14 knockdown via western blot assays (Fig. 7b).

LY294002, a chemical inhibitor of PI3K, which has been previously reported to block PI3K/Akt signaling [34]. Here, we found that p-Akt expression levels was significantly reduced in LY294002 treated group (Additional file 3: Fig. S5a). Moreover, the abilities of migration and invasion in HCT116 and HCT8 cells treated with LY294002 were significantly decreased in comparison with that in control group (Additional file 3: Fig. S5b, c). Taken together, we concluded that METTL14 exerted its biological function via inactivating SOX4 mediated PI3K/Akt pathway.

## Discussion

Numerous layers of epigenetic modulation that arise from modification of DNA and proteins have been well studied, but RNA modification are still elusive [3]. Similar to DNA modification, over 100 types of post-transcriptional modifications have been identified in all RNA species. Among these modifications, RNA m6A modification account for more than 80% of RNA modification, and have been reported to be play significant roles in pre-mRNA splicing, miRNA processing, translation regulation and mRNA decay [35, 36]. METTL14, acting as the central component of N6-methytransferse complex, has been verified to be dysregulated and involved in the initiation and progression of various malignancies. In the present study, we unveiled that METTL14 was significantly downregulated in CRC tissues, and deceased METTL14 was accompanied by a poor CRC prognosis. Subsequently, to identify the reason for low METTL14 in CRC, we analyzed the ChIP-Seq results from ENCODE database, we focused on H3K4me3 and proved that KDM5C-mediated demethylation of H3K4me3 inhibited METTL14 transcription and lead to the suppression of METTL14 in CRC through ChIP and western blot assays. Then we found that METTL14 was significantly downregulated in CRC cell lines, especially in HCT116 and HCT8 cell lines, and we selected HCT116 and HCT8 cell lines with lower METTL14 for follow-up experiments. Suppression of METTL14 markedly promoted the ability of migration and invasion, whereas overexpression of METTL14 suppressed the ability of migration and invasion in HCT116 and HCT8 cells. Similarly, in vivo experiments indicated that METTL14 knockdown promoted HCT116 cells metastasis, while METTL14 upregulation inhibited HCT116 cells metastasis. These results confirmed that METTL4 function as a tumor suppressor in CRC.

To further address the role of METTL14, we combined the data from RNA-Seq and MeRIP-Seq to reveal that SOX4 might be the downstream target of METTL14, and the m6A enrichment region of SOX4 located around the stop codon. SOX4 was negatively regulated by METTL14 and modified by METTL14-intermediated m6A methylation as detected using MeRIP-qPCR and luciferase reporter assays.

M6A reader proteins (IGF2BP1/2/3, eIF3, YTHDF1/2/3 and so on) can bind to m6A modified motif indirectly or directly to affect RNA function [37]. Here, we found that YTHDF2 knockdown could augment the SOX4 expression in CRC cells. Through RNA stability assay, Half-life of SOX4 mRNA in METTL14 stable knockdown HCT116 cells was found to be significantly longer than that in control cells, m6A modification could trigger mRNA degradation via the m6A reader protein YTHDF2 [38], and we also detected that YTHDF2 could bind to SOX mRNA through YTHDF2-RIP assay. These data indicated that SOX4 was a target of YTHDF2 in CRC.

SOX4 is a prominent tumor-related transcription factor and its expression is increased in multitude of human cancers, and has been demonstrated to participant in the TGF-β induced EMT, a process closely associated with increases in migrative and invasive capacity, in metastasis and in chemotherapy drug resistance [14, 39, 40]. Moreover, SOX4 was reported to be involved in many pathways that are commonly activated in various cancers, including PI3K/Akt signaling [41], Wnt signaling [42, 43] and MAPK signaling [44]. In this study, we validated the mRNA and protein levels of SOX4 were significantly upregulated in CRC tissues. Depletion of SOX4 could markedly suppress the ability of migration and invasion, while the inhibitory effect caused by METTL14 overexpression could be reversed by SOX4 upregulation in CRC cells, and the results of in vivo experiments were in agreement with those in vitro experiments. Furthermore,to better understand the underlying molecular mechanism of METTL14 in CRC, on the one hand, we found that METTL14 knockdown could elevate the expression of Vimentin and N-cadherin, and decrease the expression of E-cadherin, in other words, METTL14 knockdown facilitates EMT process in CRC, and this promption could be reversed by the depletion of SOX4, on the other hand, we noticed that loss of METTL14 could activate PI3K/Akt signaling, and this activation was abrogated by disruption of SOX4 in CRC. LY294002, a chemical inhibitor of PI3K, could obviously inactivate PI3K/Akt signaling as well as impair the ability of the migration and invasion in HCT116 and HCT8 cells. Taken together, these results indicated inhibition of METTL14 in CRC promoted SOX4-mediated EMT process and activated SOX4-mediated PI3K/Akt signaling pathway.

In summary, our current work elucidated the key role of METTL14-mediated m6A modification in human CRC progression and a charmingm6A-dependent regulatory mechanism. We demonstrated that METTL14 epigenetically inhibited the expression of SOX4 via an m6A-YTHDF2-dependent mechanism. The discovery of the METTL14/SOX4 axis and its impact on CRC metastasis will aid in further CRC study and in exploring efficient therapeutic strategies against CRC.

## Supplementary information

**Additional file 1: Table S1.** The sequence of primers. **Table S2.** The sequence of shRNAs. **Table S3**. The sequence of primers for ChIP. **Table S4**. Antibodies for western blot, ChIP and IHC

**Additional file 2.** Supplementary materials and methods.

**Additional file 3:Figure S1.** KDM5C was upregulated and negatively correlated with METTL14 expression. a. KDM5C expression in CRC tissues and normal tissues from TCGA cohort. b. qRT-PCR was used to detect the expression of KDM5C mRNA in CRC tissues and matched adjacent tissues. c. The correlation between KDM5C and METTL14 in CRC tissues. ***P < 0.001. **Figure S2.** The expression of SOX4 mRNA in the GSE9348, GSE44076 and GSE41657. **P < 0.01, ***P < 0.001. **Figure S3.** YTHDF1/3 have no effect on SOX4. a. The expression of SOX4 mRNA were detected using qRT-PCR after YTHDF1/3 knockdown in HCT116 and HCT8 cells. b. The expression of SOX4 protein were detected using western blot after YTHDF1/3 knockdown in HCT116 and HCT8 cells. c. The expression of YTHDF2 was detected in 30 CRC tissues and matched ANTs using qRT-PCR. ***P < 0.001. **Figure S4.** SOX4 served as an oncogene and reversed the effects of METTL14 in CRC. a. Representive amages of transwell migration in sh-NC and sh-SOX4 groups. b. Representive amages of transwell invasion in sh-NC and sh-SOX4 groups. c. Representive amages of transwell migration in indicated groups. d. Representive amages of transwell invasion in indicated groups. **Figure S5.** LY294002 could inactivate PI3K/Akt signaling as well as impair the ability of the migration and invasion in HCT116 and HCT8 cells. a. Protein levels of Akt and p-Akt were detected by western blot in HCT116 and HCT8 cells with indicated treatment. b,c. Transwell migration(b) and invasion(c) assays were employed to detect the invasive abilities of HCT116 and HCT8 cells with indicated treatment. **P < 0.01.

**Additional file 4: Table S1-S2.**

## Data Availability

The datasets used and/or analyzed during the current study are available from the corresponding author on reasonable request.

## Abbreviations

ChIP: Chromatin immunoprecipitation
CRC: Colorectal cancer
CI: Confidence interval
ENCODE: Encyclopedia of DNA Elements
KDM5C: Lysine-specific histone demethylase 5C
H3K4me3: Histone H3 lysine 4 tri-methylation
HE: Hematoxylin and eosin
IHC: Immunohistochemistry
qRT-PCR: Quantitative reverse transcription polymerase chain reaction
m6A: N6-methyladenosine
METTL14: Methyltransferase-like 14
METTL3: Methyltransferase-like 3
YTHDF1/2/3: YTH domain-containing family protein 1/2/3
SOX4: SRY-related high-mobility-group box 4
MeRIP-Seq: m6A-RNA immunoprecipitation sequencing
RNA-Seq: Transcrptomic RNA sequencing
RIP: RNA immunoprecipiation
MeRIP: m6A-RNA Immunoprecipitation
