# Supplementary material for: METTL14-mediated N6-methyladenosine modification of SOX4 mRNA inhibits tumor metastasis in colorectal cancer
Source: Mol Cancer. 2020 Jun 17;19:106. doi: 10.1186/s12943-020-01220-7 (PMC7298962; doi:10.1186/s12943-020-01220-7)
Supplement: Supplementary file 1 — Additional file 1: Table S1. The sequence of primers. Table S2. The sequence of shRNAs. Table S3. The sequence of primers for ChIP. Table S4. Antibodies for western blot, ChIP and IHC [file 12943_2020_1220_MOESM1_ESM.pdf]

Table S1. The sequence of primers

METTL14	Forward: 5′-GTT GGA ACA TGG ATA GCC GC-3′
	Reverse: 5′-CAA TGC TGT CGG CAC TTT CA-3′
YTHDF2	Forward: 5′-TAGCCAACTGCGACACATTC-3′
	Reverse: 5′-CACGACCTTGACGTTCCTTT-3′
KDM5C	Forward :5′-GAGGTGACCCTGGATGAGAA-3′
	Reverse :5′-CAGGAGCTGAGGTCTGAAC-3′
YTHDF1	Forward:5′-ATGTCGGCCACCAGCGTGGACA-3′
	Forward:5′-TCATTGTTTGTTTCGACTCTGC-3′
YTHDF3	Forward : 5′-TGACAACAAACCGGTTACCA-3′
	Reverse: 5′-TGTTTCTATTTCTCTCCCTACGC-3′
GAPDH	Forward: 5’-GGTGGTCTCCTCTGACTTCAA-3’
	Reverse: 5’-GTTGCTGTAGCCAAATTCGTTGT-3’
SOX4(precursor)	Forward:5′-GCAAGATCATGGAGCAGTCG-3′
	Reverse:5′-GGGCCGGTACTTGTAGTCG-3′
SOX4(mature)	Forward:5′-GGTCTCTAGTTCTTGCACGCTC-3′
	Reverse:5′-CGGAATCGGCACTAAGGAG- 3′

Table S2. The sequence of shRNAs

shNC	ACTCAAAAGGAAGTGACAAGA
shMETTL14-1	GCTAAAGGATGAGTTAAT
shMETTL14-2	GGACTTGGGATGATATTAT
shSOX4	AAGAAGGTGAAGCGCGTCTA
shYTHDF2-1	GAACGTCAAGGTCGTGGGAAA
sh-YTHDF2-2	ACACATTCGCCTAGAGAACAA
shYTHDF1-1	GATACAGTTCATGACAATGA
shYTHDF1-2	GAAACGTCCAGCCTAATTCT
shYTHDF3-1	GGACGTGTGTTTATAATTA
shYTHDF3-2	GACTAGCATTGCAACCAAT
shKDM5C-1	GGAGGAAGGTGGTTATGAA
shKDM5C-2	GGAGGAAGGTGGTTATGAA

Table S3. The sequence of primers for ChIP.

C1	Forward: 5’-AACAGACGCCATTCACTAT- 3′
	Reverse:5′-AGGACTGGTAGCCATCCTG- 3′
C2	Forward: 5’-GAAGCAGAGGGTAGGTGAG- 3′
	Reverse:5′-ATGGCAAGCACTGTGATAGG- 3′
C3	Forward: 5’-AAATACACTTTAACTCATATGGACA- 3′
	Reverse:5′-GGGCAAGACTAGATTCTACTTAG- 3′
C4	Forward: 5’-CGCCACTGTAGGCTTGAAATA- 3′

	Reverse:5′-AACCCAAGGATAAGGAAACAAA- 3′
C5	Forward: 5’-TGTGGCTGTTACCTGCGTGA- 3′
	Reverse: 5’-AGCGGTGTAGAAGTGCTTGTTATT- 3′
C6	Forward: 5’-AAACGTCAGGACTGTCATCT- 3′
	Reverse: 5’-GCAGGTTCCCTTGGTGTAT- 3′
C7	Forward: 5’-TCCAAGTGGCGTCACATAGC- 3′
	Reverse: 5’-CCGGGAGCTTACGAACAGAG- 3′
C8	Forward: 5’-TTTCTGGTCCTGCGATCTTG- 3′
	Reverse: 5’- ATCCTGGCATTCGGTTTAGC- 3′

Table S4. Antibodies for western blot, ChIP and IHC

**Antibody name**	**Source**	**Item number**
METTL14	Abcam	ab46154
SOX4	AVIVA	ARP33252_P050
KDM5C	CST	#3876
YTHDF1	proteintech	17479-1-AP
YTHDF2	proteintech	24744-1-AP
YTHDF3	proteintech	25537-1-AP
M6A	Abcam	ab208577
H3K4me3	proteintech	39159
Akt	CST	#9272
phospho(Ser473)-Akt	CST	#4060
PI3K	CST	#4249
phospho(Tyr458)-PI3K	CST	#4228
GAPDH	proteintech	10494-1-AP
