# Supplementary material for: METTL14-mediated N6-methyladenosine modification of SOX4 mRNA inhibits tumor metastasis in colorectal cancer
Source: Mol Cancer. 2020 Jun 17;19:106. doi: 10.1186/s12943-020-01220-7 (PMC7298962; doi:10.1186/s12943-020-01220-7)
Supplement: Supplementary file 2 — Additional file 2. Supplementary materials and methods. [file 12943_2020_1220_MOESM2_ESM.pdf]

Supplementary materials and methods

Western blot

Total protein of CRC cells were extracted using RIPA lysis buffer supplemented with phosphatase and protease inhibitor, then the protein samples were separated on a 10% SDS-PAGE and then transferred to PVDF membranes(Millipore, Billerica, MA, USA). After blocking with 3% nonfat milk in TBST for 1 h at room temperature, the membranes were then incubated with primary antibody at 4℃ overnight. HRP-conjugated goat anti-rabbit secondary antibody were employed to detect the protein of interest. The information of antibodies were listed in Additionally file 1:Table S4.

Immunohistochemistry(IHC) analysis

Tissue arrays were constructed using 136 pairs of CRC tissues and matched ANTs. Immunohistochemical staining was employed on 5-mm sections of paraffin-embedded human CRC tissues and matched ANTs to detect the expression of METTL14 and SOX4. In brief, the slides were incubated with anti-METTL14 and anti-SOX4 overnight. Subsequent steps were performed using Universal Daka LSAB + kit and Peroxidase (LASB + Kit, HRP) in accordance with the manufacturer's instruction (DAKO). The intensity of staining was scored by two independent pathologists in the following four categories: no staining=0, weak staining=1, moderate staining=2 and strong staining=3. The stain-positive sections were categorized into four grades: 0 (0%), 1 (1-33%), 2 (34%-66%), and 3 (67%-100%). The final ICH score was calculated by multiplying the percentage of positive cells with the intensity score.

RNA m6A dot blot assays

The poly(A)+RNAs were firstly denatured via heating at 65 ℃ for 5min and transferred onto a nitrocellulose membrane(GE Healthcare, USA). Then the membrane were UV cross-linked, blocked, and incubated with m6A antibody(1:1000, abcam，USA）overnight at 4℃ and subsequently incubated with HRP-conjugated goat anti-mouse IgG(1:5000, Abcam, USA) for 1 h. Lastly, the membranes were visualized using the chemiluminescence system(Bio-Rad, USA). The membrane

stained with 0.02% methylene blue(MB) in 0.3M sodium acetate(PH5.2), was used to ensure consistency among different groups.

RNA immunoprecipitation (RIP) assay

An RIP assay was performed with a Magna RIP™ RNA-Binding Protein Immunoprecipitation Kit (Millipore, Billerica, USA) in accordance with the manufacturer's protocol. Cells were lysed in RIP lysis buffer, and then, 100 µl whole-cell extract was incubated with magnetic beads conjugated with anti-YTHDF2 or IgG for 6 h at 4℃. After that, the beads were incubated with proteinase K with shaking to remove protein. Finally, the coprecipitated RNAs were extracted and subjected to qRT-PCR using primers for METTL14 and normalized to input.

For the m6A RNA binding assays, the Magna MeRIP™ m6A Kit (Millipore, Billerica, USA) was used. In brief, RNAs were chemically fragmented to ~100 nt, and fragmented RNA was then incubated with magnetic beads conjugated with m6A antibody (Millipore, Billerica, USA) for immunoprecipitation. The enrichment of m6A-containing mRNA was then analyzed through qRT-PCR and normalized to input.

RNA-Seq and MeRIP-Seq

For RNA-Seq, Total RNA from CRC cells with stable METTL14 knockdown and their corresponding cells were extracted using Trizol reagent(Invitrogen, USA). An Agilent 2100 Bioanaluzer(Agilent, CA, USA) and NanoDrop 2000(Thermo Fisher, MA, USA) were employed to analyze the total RNA quality and quantity. Total RNA was treated with the Epicentre Ribo-Zero kit to remove the all the rRNAs. The remaining RNAs were processed using the TruSeq RNA sample Prep Kit according to the illumina protocol. Then, RT-PCR was performed with Phusion High-Fidelity DNA polymerase, Index (X) Primer, and Universal PCR primers. Finally, the products were purified using the AMPure XP system, and library quality was evaluated on an Agilent Bioanalyzer 2100 system. The RNA library was sequenced on an Illumina Hiseq 4000 platform, and 150 bp paired-end reads were generated.

For MeRIP-Seq, mRNA was further purified using the NEBNext Poly(A) mRNA Magnetic Isolation Kit (NEB, UK). RNA fragmentation was performed by incubation

with Magnesium ions at 94°C using NEBNext Magnesium RNA Fragmentation Module (NEB, UK). m6A-IP and library preparation were performed per the reported protocol with some modifications. Briefly, 1 ug fragmentated mRNA was mixed with 6.5 ug m6A antibody (Abcam, UK) in 1x IP buffer and incubated with head-to-tail mixing at 4℃ for 2 h. The mixture was supplemented with 50 mL Dynabeads Protein A (Thermo Fisher, MA, USA) prewashed two times with 1x IP buffer and incubated with head-to-tail mixing at 4℃ for another 2 h. The beads were then separated and washed with 1x IP buffer three times before eluted with m6A elution buffer for two times. The eluates were recovered with 500uL ethanol which added 20 µl of 3 M stock of NaOAc. The enriched mRNA fragments were then used to construct libraries with VAHTS Total RNA-seq (H/M/R) Library Prep Kit for Illumina (Vazyme, CN). Sequencing was carried out on Illumina HiSeq 2500 with paired-end 150bp read length.

Luciferase Reporter Assay

pmirGLO luciferase expression vector(Promega) was employed to construct the reporter plasmid, which contained both a firefly luciferase(F-Luc) and a Renilla luciferase(R-Luc). Wild-type SOX4 reporter plasmid was cloned by inserting the full-length of SOX4 transcript after the Fluc coding sequence. The mutant SOX4 reporter plasmid was made by replacing the adenosine bases within the m6A consensus sequences to cytosine. Cells seeded in 6-well plate were transfected with 500ng of wild-type and mutated F-Luc-SOX4 fusion reporter plasmid. After 48h, cells were assayed with Dual-GLO Luciferase system (Promega). F-Luc activity was used to assess the effect of m6A modification on SOX4 expression. R-Luc was used to normalize the transfection efficiency of the reporter plasmid.
