# Supplementary figures and images for: METTL14-mediated N6-methyladenosine modification of SOX4 mRNA inhibits tumor metastasis in colorectal cancer
Source: Mol Cancer. 2020 Jun 17;19:106. doi: 10.1186/s12943-020-01220-7 (PMC7298962; doi:10.1186/s12943-020-01220-7)

**Fig S1**

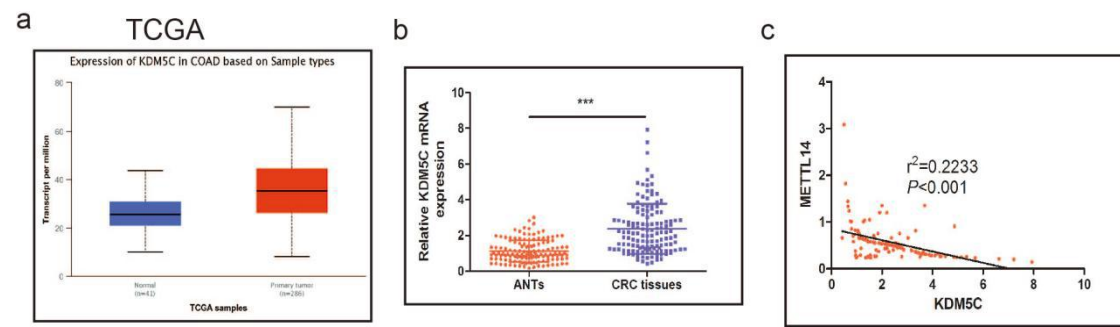

**Fig S2**

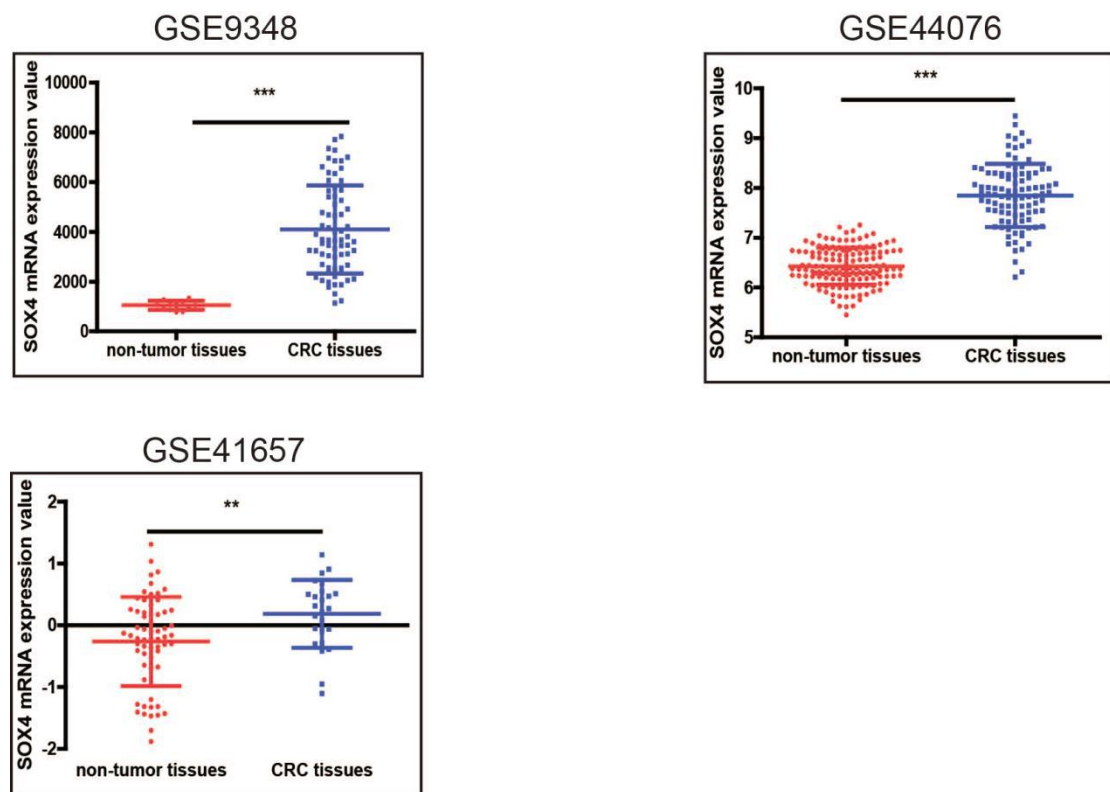

**Fig S3**

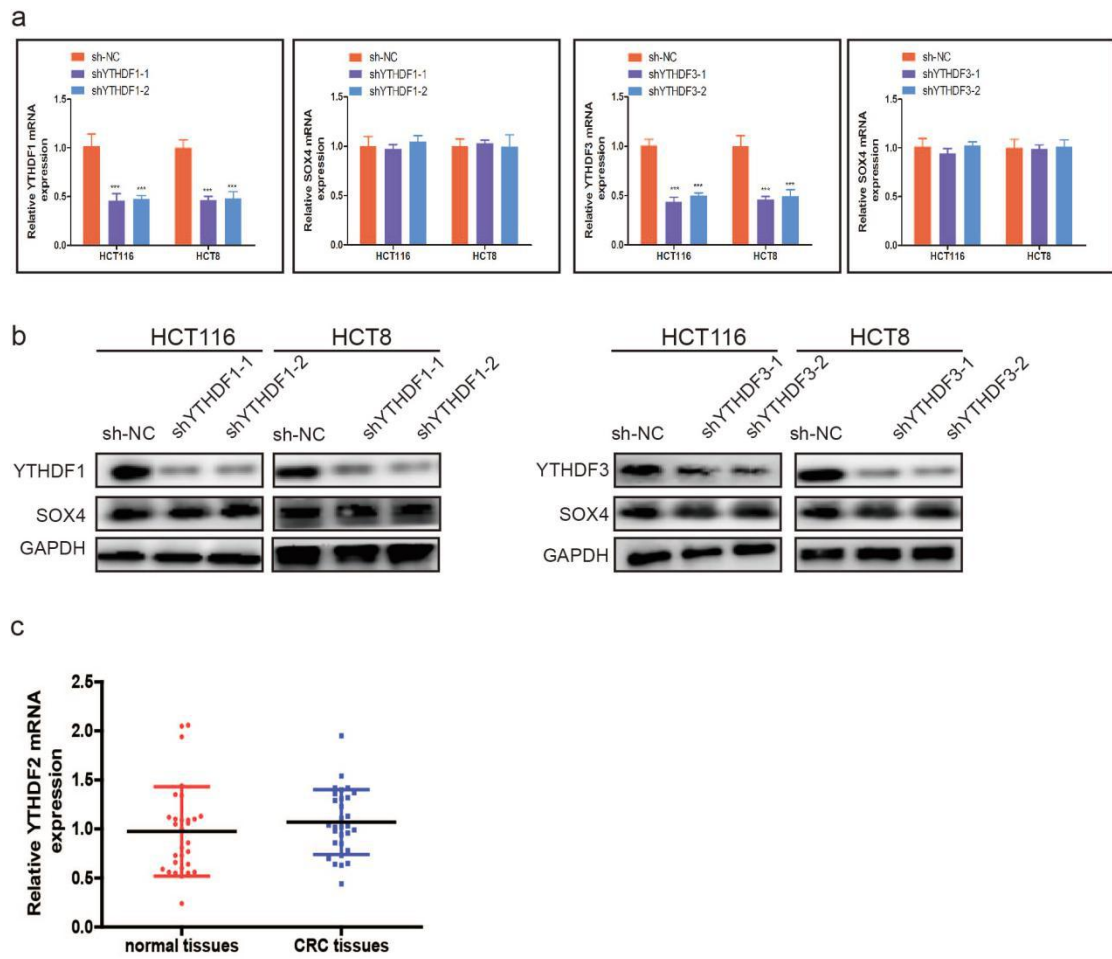

Fig S4

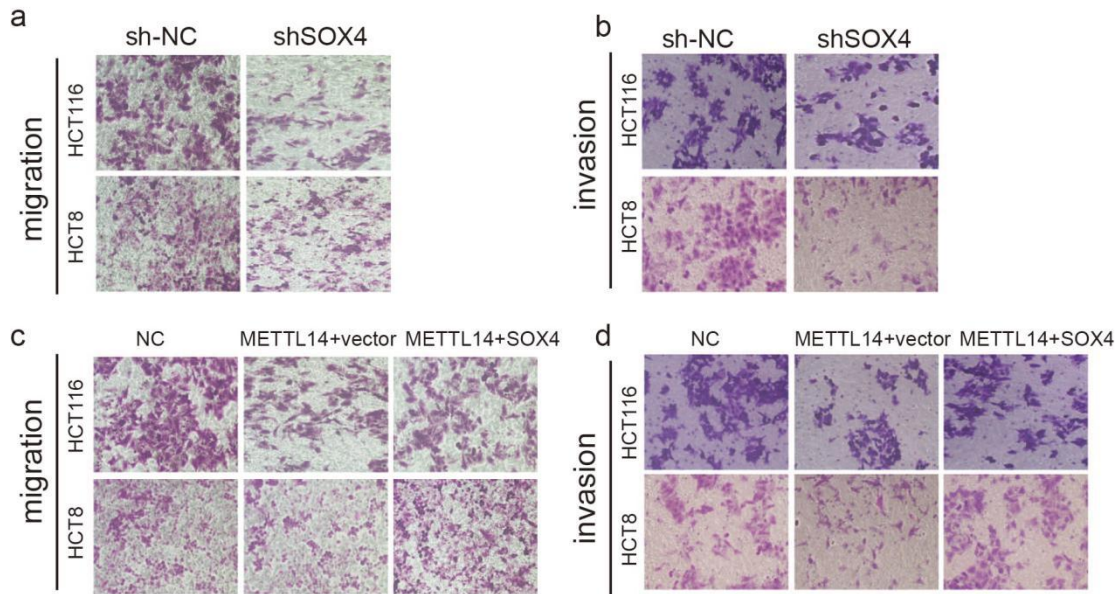

**Fig S5**

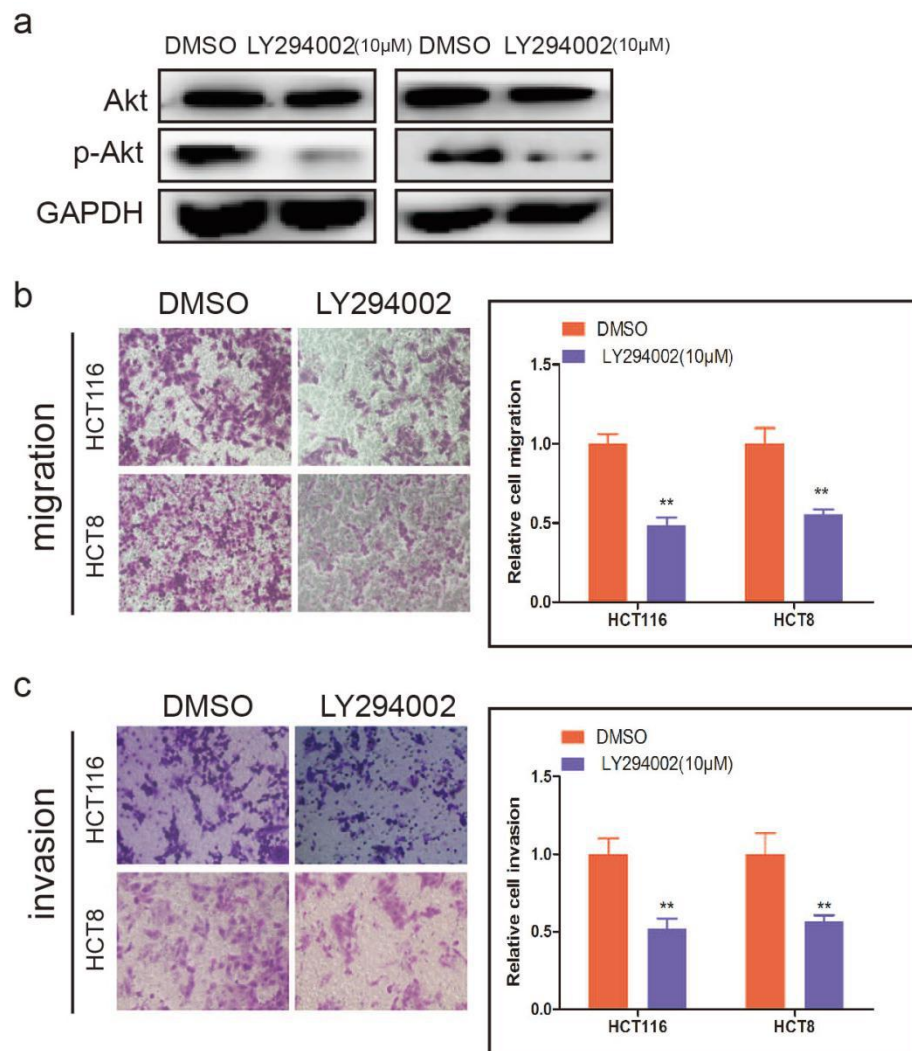

Supplement: Supplementary file 3 — Additional file 3:Figure S1. KDM5C was upregulated and negatively correlated with METTL14 expression. a. KDM5C expression in CRC tissues and normal tissues from TCGA cohort. b. qRT-PCR was used to detect the expression of KDM5C mRNA in CRC tissues and matched adjacent tissues. c. The correlation between KDM5C and METTL14 in CRC tissues. ***P < 0.001. Figure S2. The expression of SOX4 mRNA in the GSE9348, GSE44076 and GSE41657. **P < 0.01, ***P < 0.001. Figure S3. YTHDF1/3 have no effect on SOX4. a. The expression of SOX4 mRNA were detected using qRT-PCR after YTHDF1/3 knockdown in HCT116 and HCT8 cells. b. The expression of SOX4 protein were detected using western blot after YTHDF1/3 knockdown in HCT116 and HCT8 cells. c. The expression of YTHDF2 was detected in 30 CRC tissues and matched ANTs using qRT-PCR. ***P < 0.001. Figure S4. SOX4 served as an oncogene and reversed the effects of METTL14 in CRC. a. Representive amages of transwell migration in sh-NC and sh-SOX4 groups. b. Representive amages of transwell invasion in sh-NC and sh-SOX4 groups. c. Representive amages of transwell migration in indicated groups. d. Representive amages of transwell invasion in indicated groups. Figure S5. LY294002 could inactivate PI3K/Akt signaling as well as impair the ability of the migration and invasion in HCT116 and HCT8 cells. a. Protein levels of Akt and p-Akt were detected by western blot in HCT116 and HCT8 cells with indicated treatment. b,c. Transwell migration(b) and invasion(c) assays were employed to detect the invasive abilities of HCT116 and HCT8 cells with indicated treatment. **P < 0.01. [file 12943_2020_1220_MOESM3_ESM.pdf]
